# Supplementary material for: ZIC3 Controls the Transition from Naive to Primed Pluripotency
Source: Cell Rep. 2019 Jun 11;27(11):3215–3227.e6. doi: 10.1016/j.celrep.2019.05.026 (PMC6581693; doi:10.1016/j.celrep.2019.05.026)
Supplement: Document S1. Figures S1–S9 [file mmc1.pdf]

Cell Reports, Volume *27*

Supplemental Information

ZIC3 Controls the Transition from Naive to Primed Pluripotency

Shen-Hsi Yang, Munazah Andrabi, Rebecca Biss, Syed Murtuza Baker, Mudassar Iqbal, and Andrew D. Sharrocks

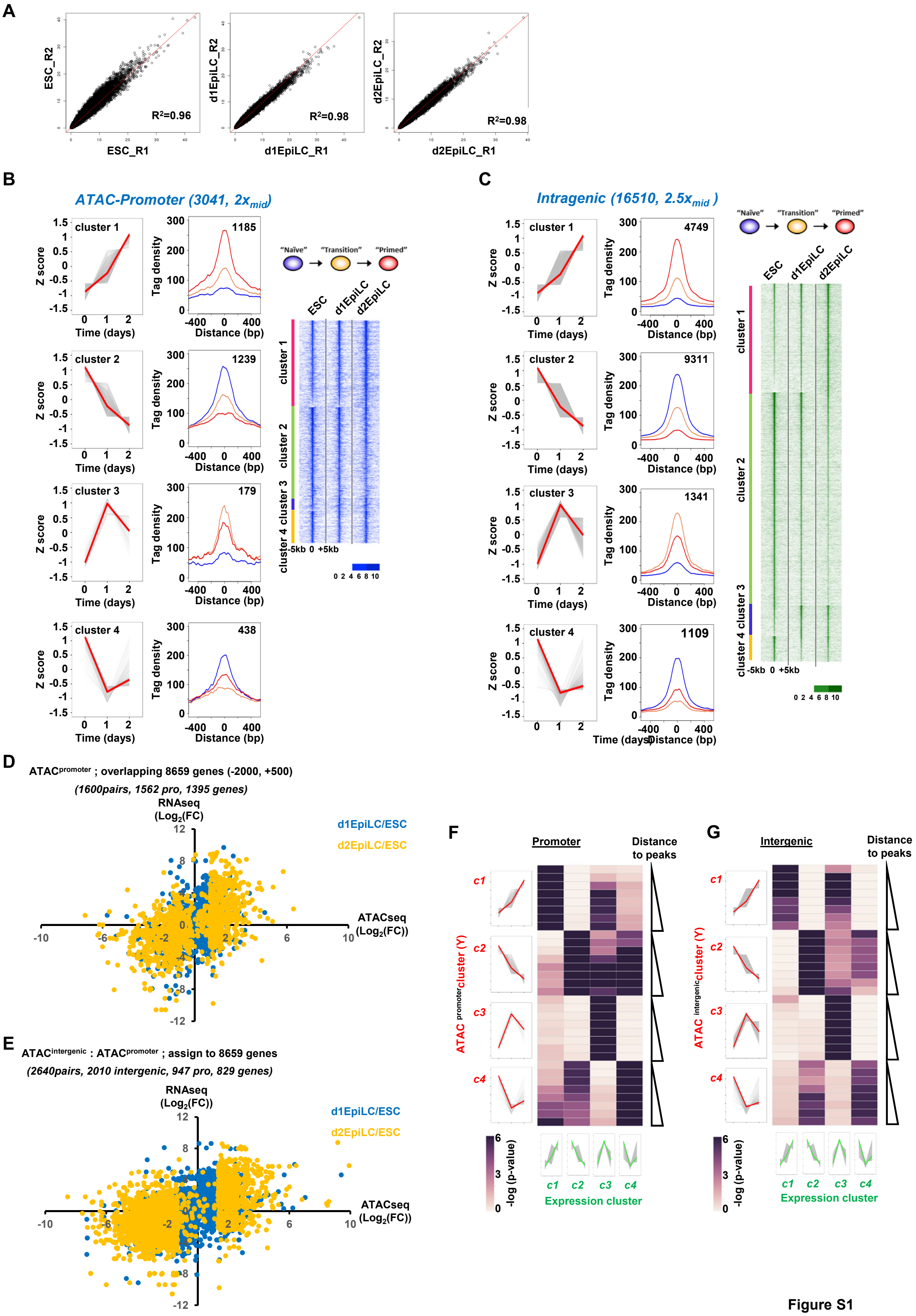
A
ESC_R2
ESC_R1
R²=0.96
d1EpiLC_R2
d1EpiLC_R1
R²=0.98
d2EpiLC_R2
d2EpiLC_R1
R²=0.98
B
ATAC-Promoter (3041, 2x mid)
cluster 1
cluster 2
cluster 3
cluster 4
Z score
Time (days)
Tag density
Distance (bp)
1185
1239
179
438
"Naïve"
"Transition"
"Primed"
ESC
d1EpiLC
d2EpiLC
-5kb 0 +5kb
0 2 4 6 8 10
C
Intragenic (16510, 2.5x mid)
4749
9311
1341
1109
D
ATACpromoter ; overlapping 8659 genes (-2000, +500)
(1600pairs, 1562 pro, 1395 genes)
RNAseq
(Log2(FC))
ATACseq
(Log2(FC))
d1EpiLC/ESC
d2EpiLC/ESC
E
ATACintergenic : ATACpromoter ; assign to 8659 genes
(2640pairs, 2010 intergenic, 947 pro, 829 genes)
F
Promoter
Distance
to peaks
ATAC promoter cluster (Y)
c1
c2
c3
c4
-log (p-value)
Expression cluster
G
Intergenic
ATAC intergenic cluster (Y)

Figure S1

Figure S1. Open chromatin profiling during the ESC to EpiLC transition. Related to Figure 1. (A) Scatter plots of ATAC-seq data from two independent biology repeats (R1 and R2) from ESCs, d1EpiLCs and d2EpiLCs. The numbers of reads from the R1 and R2 repeats at each time point were determined in a consolidated peak set from all time points (362,008 peaks). R2 values are shown. (B and C) Heat maps of the ATAC-seq profiles across a 10 kb window of promoter regions (-2 to +0.5 kb relative to the TSS)(B) and intragenic regions (C) showing >2 fold (promoters) or >2.5 fold (intragenic regions) change in accessibility between any two conditions (right). Average tag densities of each of four identified clusters (middle; blue=ESC, orange=d1EpiLCs, red=d2EpilCs) and average tag density profiles (z scored and medians [red] and standard deviation [grey] shown) across the time course (left) are shown. (D and E) Scatter plots comparing ATAC-seq signals from individual peaks to the RNA-seq signals associated with their associated genes. Data are plotted as $\log_2$ fold change between two conditions (blue for d1EpiLCs versus ESCs and orange for d2EpiLCs versus ESCs). Promoter-located ATAC-seq peaks (D) or intergenic located ATAC-seq peaks (E) were analysed. ATAC-peaks showing differentially accessibility between any two time points were assigned to 8659 differentially expressed genes (defined as >1.5 fold change between any time point) by 1 bp overlap with their TSS (for promoter-located ATAC-seq peaks) or by first locating the closest differentially changing ATAC-seq peak at a promoter and then identifying the associated gene by 1 bp overlap with a TSS (for intergenic-located ATAC-seq peaks). The total number of ATAC-seq peak pairs to promoters (pro) and genes is indicated. Pearson's correlations are 0.428 (d1EpiLC) and 0.502(d2EpiLC) (D), 0.424(d1EpiLC) and 0.538(d2EpiLC) (E). (F and G) Enrichment of genes associated with each ATAC-seq peaks cluster, (F; promoter and G; intergenic)(illustrated on the left), and each gene expression based cluster (illustrated on the bottom). These enrichments are calculated at different peak-to-gene genomic distances (+/- 10, 25, 50, 100, 125, 150, 200 and 250 kb; increasing distances illustrated by a triangle). The colours represent the p-values calculated from a hypergeometric model.

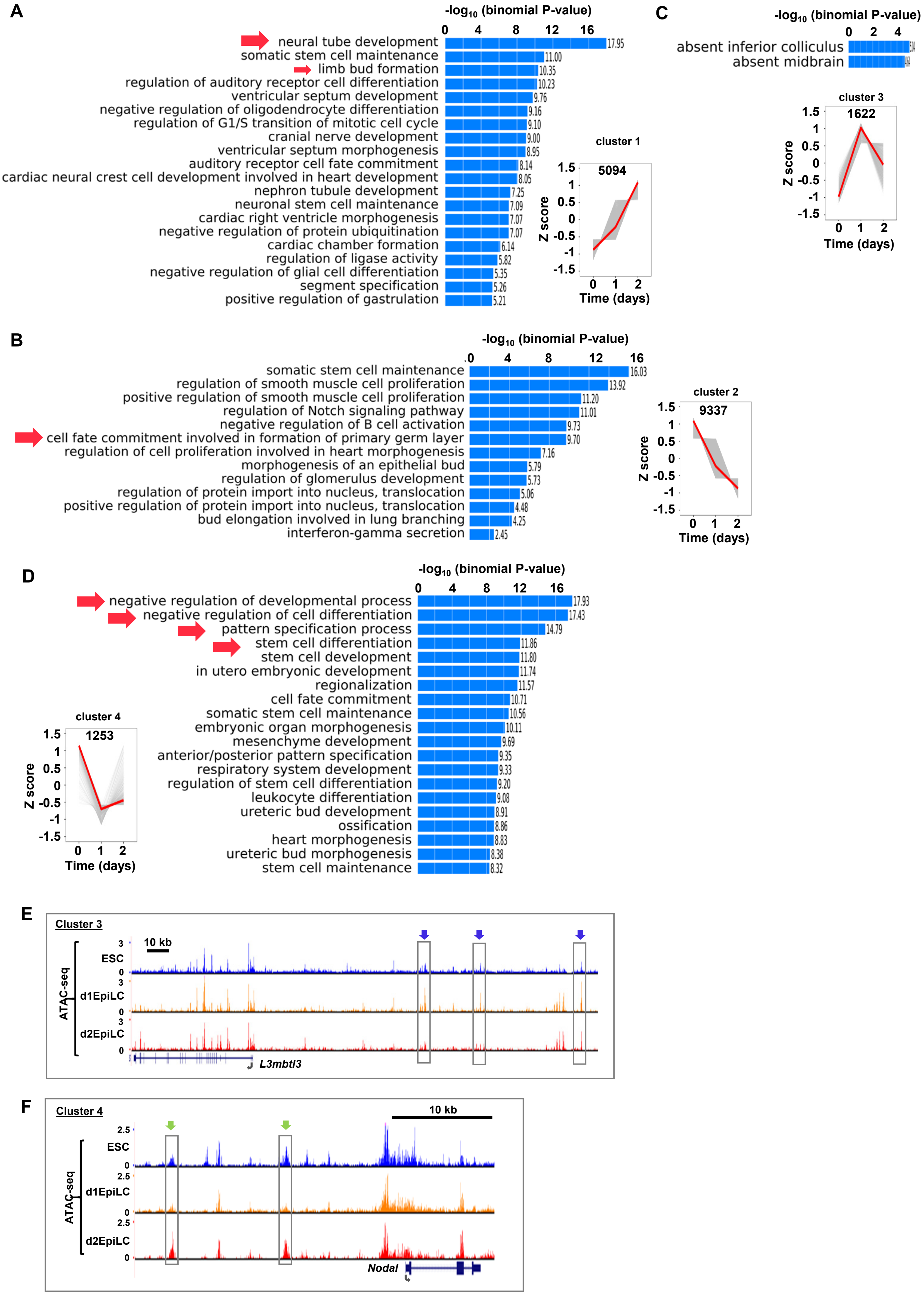
A
-log10 (binomial P-value)
0 4 8 12 16
neural tube development 17.95
somatic stem cell maintenance 11.00
limb bud formation 10.35
regulation of auditory receptor cell differentiation 10.23
ventricular septum development 9.76
negative regulation of oligodendrocyte differentiation 9.16
regulation of G1/S transition of mitotic cell cycle 9.10
cranial nerve development 9.00
ventricular septum morphogenesis 8.95
auditory receptor cell fate commitment 8.14
cardiac neural crest cell development involved in heart development 8.05
nephron tubule development 7.25
neuronal stem cell maintenance 7.09
cardiac right ventricle morphogenesis 7.07
negative regulation of protein ubiquitination 7.07
cardiac chamber formation 6.14
regulation of ligase activity 5.82
negative regulation of glial cell differentiation 5.35
segment specification 5.26
positive regulation of gastrulation 5.21
cluster 1
5094
Z score
Time (days)
C
-log10 (binomial P-value)
0 2 4
absent inferior colliculus
absent midbrain
cluster 3
1622
Z score
Time (days)
B
-log10 (binomial P-value)
0 4 8 12 16
somatic stem cell maintenance 16.03
regulation of smooth muscle cell proliferation 13.92
positive regulation of smooth muscle cell proliferation 11.20
regulation of Notch signaling pathway 11.01
negative regulation of B cell activation 9.73
cell fate commitment involved in formation of primary germ layer 9.70
regulation of cell proliferation involved in heart morphogenesis 7.16
morphogenesis of an epithelial bud 5.79
regulation of glomerulus development 5.73
regulation of protein import into nucleus, translocation 5.06
positive regulation of protein import into nucleus, translocation 4.48
bud elongation involved in lung branching 4.25
interferon-gamma secretion 2.45
cluster 2
9337
Z score
Time (days)
D
-log10 (binomial P-value)
0 4 8 12 16
negative regulation of developmental process 17.93
negative regulation of cell differentiation 17.43
pattern specification process 14.79
stem cell differentiation 11.86
stem cell development 11.80
in utero embryonic development 11.74
regionalization 11.57
cell fate commitment 10.71
somatic stem cell maintenance 10.56
embryonic organ morphogenesis 10.11
mesenchyme development 9.69
anterior/posterior pattern specification 9.35
respiratory system development 9.33
regulation of stem cell differentiation 9.20
leukocyte differentiation 9.08
ureteric bud development 8.91
ossification 8.86
heart morphogenesis 8.83
ureteric bud morphogenesis 8.38
stem cell maintenance 8.32
cluster 4
1253
Z score
Time (days)
E
Cluster 3
10 kb
ATAC-seq
ESC
d1EpiLC
d2EpiLC
L3mbtl3
F
Cluster 4
10 kb
ATAC-seq
ESC
d1EpiLC
d2EpiLC
Nodal

Figure S2

Figure S2. Gene ontology analysis of genes associated with clusters of dynamically changing ATAC-seq peaks. Related to Figure 1. (A-D) Gene ontology analysis of genes associated with the dynamically changing intergenic ATAC-seq peaks from clusters 1-4 (shown as insets; see Fig. 1B). Enriched terms associated with the biological process subcategory are shown. (E and F) UCSC genome browser views of the ATAC-seq profiles around representative gene loci associated with cluster 3 (*L3mbtl3*; E) and cluster 4 (Nodal; F) intergenic peaks. Dynamically changing peaks are boxed.

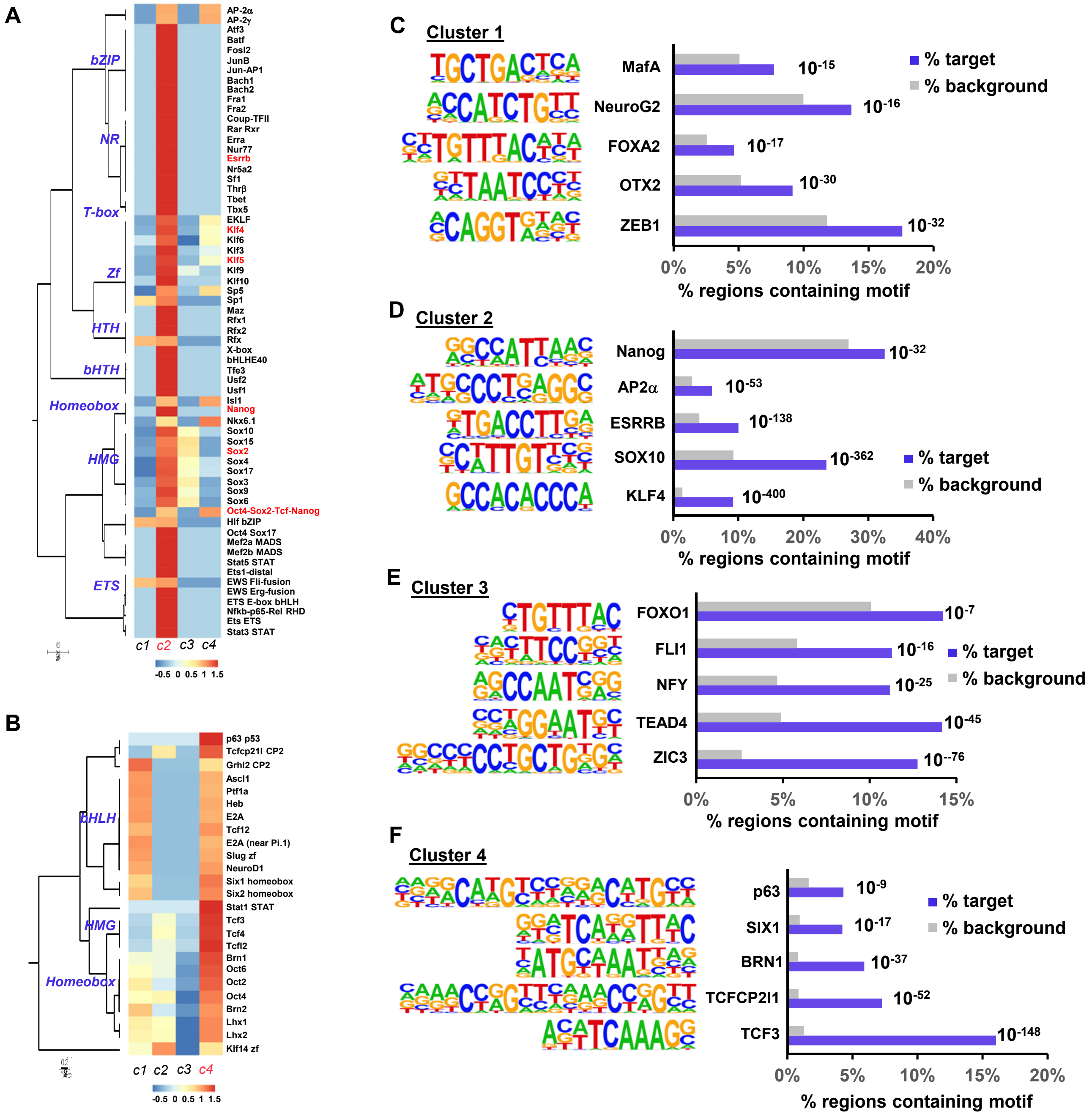

Figure S3. DNA motif enrichment in clusters of dynamically changing ATAC-seq peaks. Related to Figure 1. (A and B) Heat map showing the enrichment of transcription factor binding motifs across each of the open chromatin cluster profiles (z-normalised p-values) for motifs enriched in cluster 2 (A) or cluster 4 (B). (C-F) The top five enriched DNA motifs identified in each cluster of dynamically changing ATAC-seq peaks. The percentage of each motif present in each set of peaks and the genomic background is indicated, and the p-value is shown next to each of the columns.

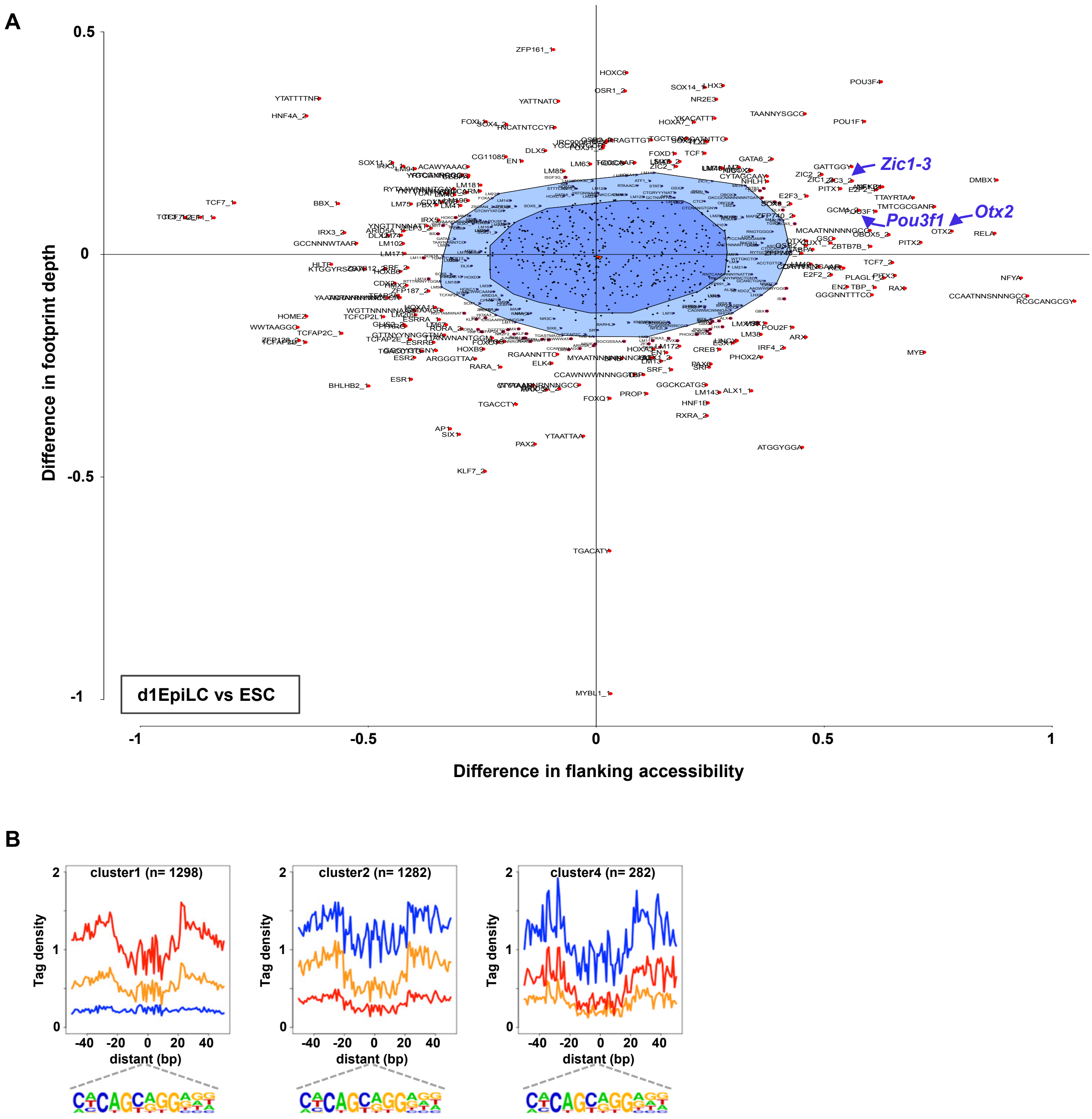

Figure S4. Dynamically changing chromatin accessibility around transcription factor binding motifs in d1EpiLCs. Related to Figure 1. (A) BagFoot analysis of the open chromatin regions in ESCs and d1EpiLCs. Motifs showing significant changes in either local accessibility and/or footprint depth are labelled. Non-significant regions are shown in the dark (bag) or light (fence) blue shaded regions. (B) Average tag densities in a 80 bp window surrounding the ZIC3 binding motif (bottom) in ESCs (blue), d1EpiLCs (orange) or d2EpilCs (red) are shown for the intergenic ATAC-seq peaks from each of the indicated clusters.

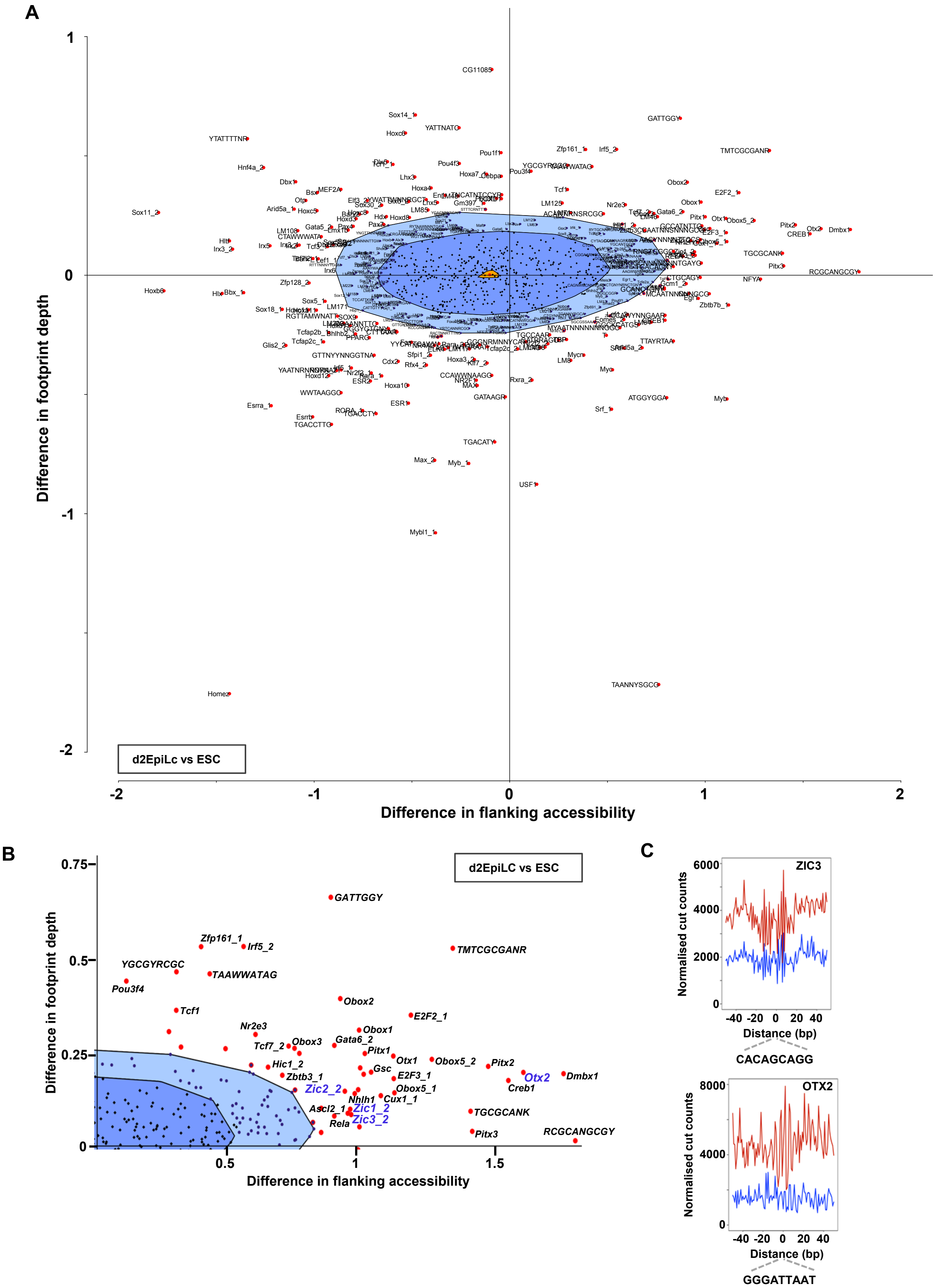

Figure S5. Dynamically changing chromatin accessibility around transcription factor binding motifs in d2EpiLCs. Related to Figure 1. (A) BagFoot analysis of the open intergenic chromatin regions in ESCs and d2EpiLCs. Motifs showing significant changes in either local accessibility and/or footprint depth are labelled. Non-significant regions are shown in the dark (bag) or light (fence) blue shaded regions. (B) Expanded version of the top right quadrant from (B). (C) Average tag densities in a 100 bp window surrounding the ZIC3 binding (top) or OTX2 (bottom) binding motifs in all peaks in ESCs (blue) or d2EpilCs (red) are shown.

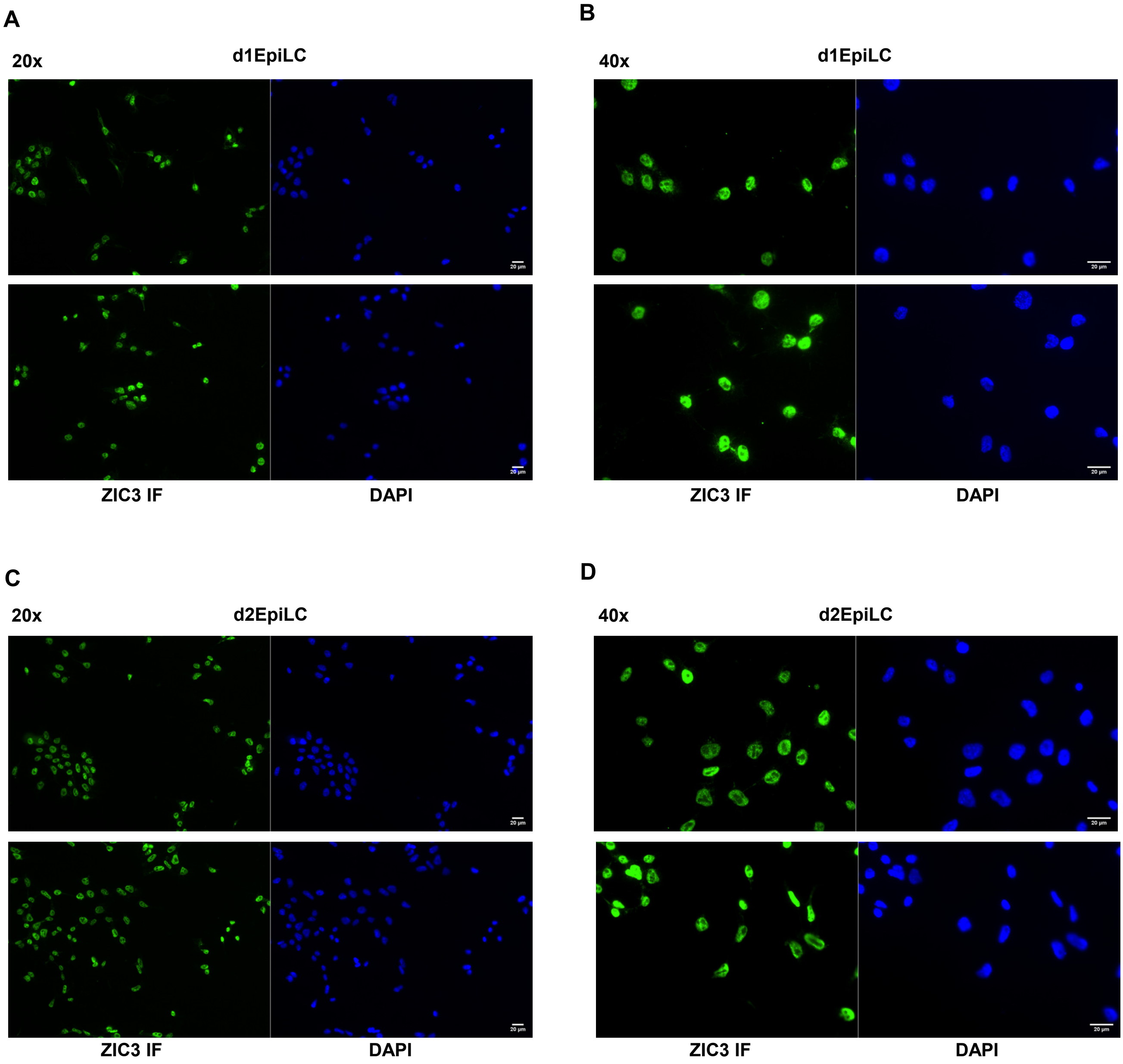

Figure S6. Immunofluorescence detection of ZIC3 in the nucleus. Related to Figure 2. ZIC3 protein was detected by immunofluorescence in d1EpiLCs (A and B) and d2 EpiLCs (C and D). Nuclei were identified by DAPI staining. Four representative fields are shown for each timepoint, with either 20x (A and C) or 40x (B and D) magnification. All cells contain nuclear ZIC3.

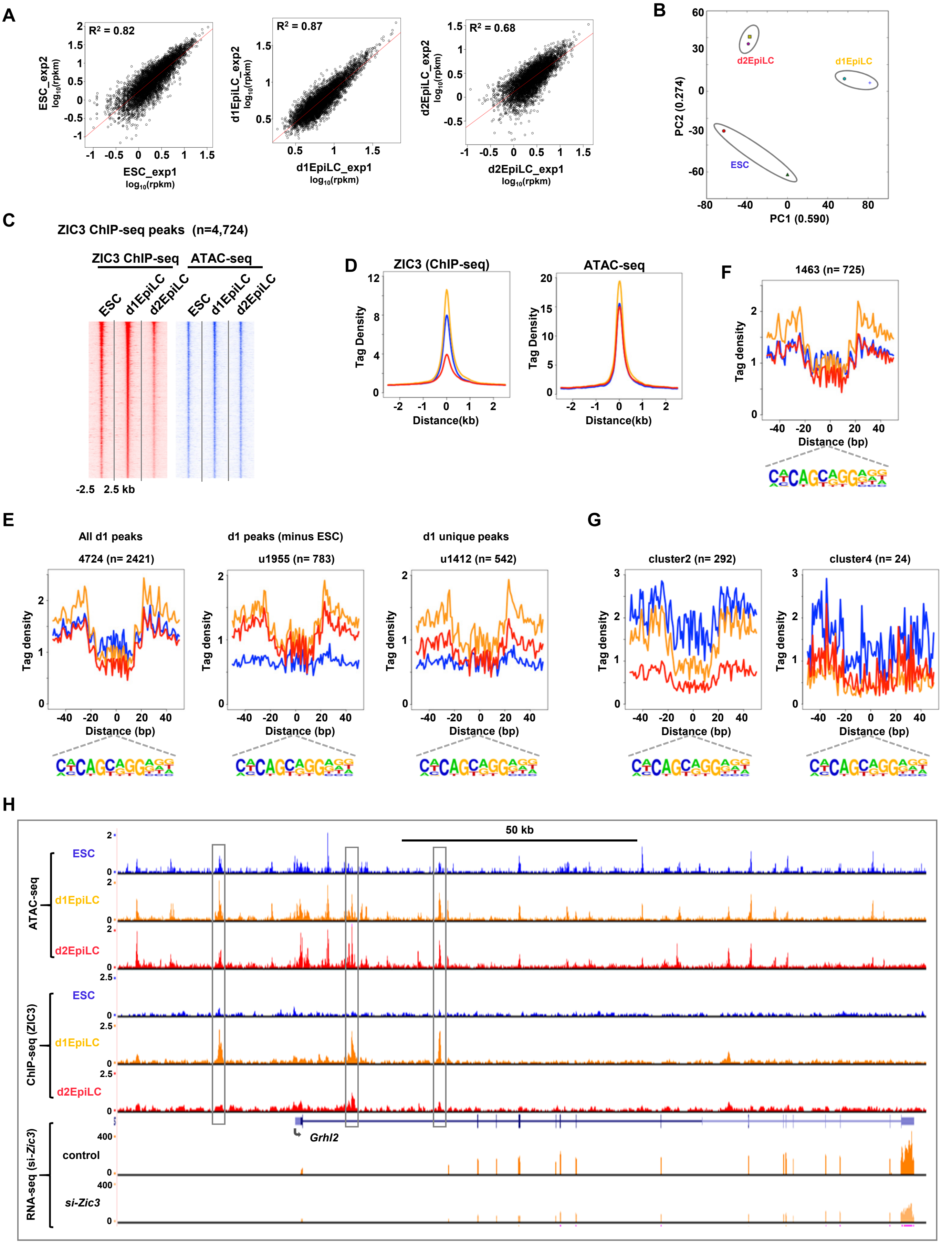
A
R² = 0.82
ESC_exp2 log10(rpkm)
ESC_exp1 log10(rpkm)
R² = 0.87
d1EpiLC_exp2 log10(rpkm)
d1EpiLC_exp1 log10(rpkm)
R² = 0.68
d2EpiLC_exp2 log10(rpkm)
d2EpiLC_exp1 log10(rpkm)
B
d2EpiLC
d1EpiLC
ESC
PC2 (0.274)
PC1 (0.590)
C
ZIC3 ChIP-seq peaks (n=4,724)
ZIC3 ChIP-seq
ATAC-seq
ESC
d1EpiLC
d2EpiLC
-2.5 2.5 kb
D
ZIC3 (ChIP-seq)
ATAC-seq
Tag Density
Distance(kb)
F
1463 (n= 725)
Tag density
Distance (bp)
E
All d1 peaks
4724 (n= 2421)
d1 peaks (minus ESC)
u1955 (n= 783)
d1 unique peaks
u1412 (n= 542)
G
cluster2 (n= 292)
cluster4 (n= 24)
H
50 kb
ATAC-seq
ChIP-seq (ZIC3)
RNA-seq (si-Zic3)
control
si-Zic3
Grhl2

Figure S7

Figure S7. ChIP-seq analysis of ZIC3 binding to chromatin in ESCs and d1EpiLCs. Related to Figure 3. (A) Scatter plots of the replicate ZIC3 ChIP-seq experiments performed in the indicated cell populations. Data are plotted based on the 4,724 high confidence ZIC3 binding regions in d1EpiLCs. Correlation coefficients (R^2) are indicated. (B) PCA analysis of the replicate ZIC3 ChIP-seq experiments performed in the indicated cell populations. (C) Heat map of the ZIC3 ChIP-seq profiles across a 5 kb window in each of the indicated cell populations (left). The corresponding ATAC-seq signals at each ZIC3 binding region are shown on the right. Data are ranked according to ZIC3 ChIP-seq signals in d1EpiLCs. (D) Average tag densities in a 5 kb window around all ZIC3 peaks (blue=ESC, orange=d1EpiLCs, red=d2EpilCs) for ZIC3 ChIP-seq signal (left) or ATAC-seq signal (right). (E-G) Average ATAC-seq tag densities in a 80 bp window surrounding the ZIC3 motif (bottom) in; (E) peaks partitioned according to their presence in d1EpiLCs (left), peaks found in d1EpiLCs but not ESCs (middle) or peaks found in d1ESCs but neither ESCs nor d2EpiLCs (right); (F) all ZIC3 ChIP-seq binding regions from clusters 1-4 in Fig. 3D; (G) ZIC3 binding regions from clusters c2 (left), or cluster c4 (right)(clusters defined in Fig. 3D). ATAC-seq tags from ESCs (blue), d1EpiLCs (orange) or d2EpilCs (red) are shown. (H) UCSC genome browser views of the ATAC-seq (top), ZIC3 ChIP-seq (middle) and RNA-seq (plus/minus *siZic3*; bottom) profiles around the entire *Grhl2* locus. Dynamically changing ZIC3 binding peaks are boxed.

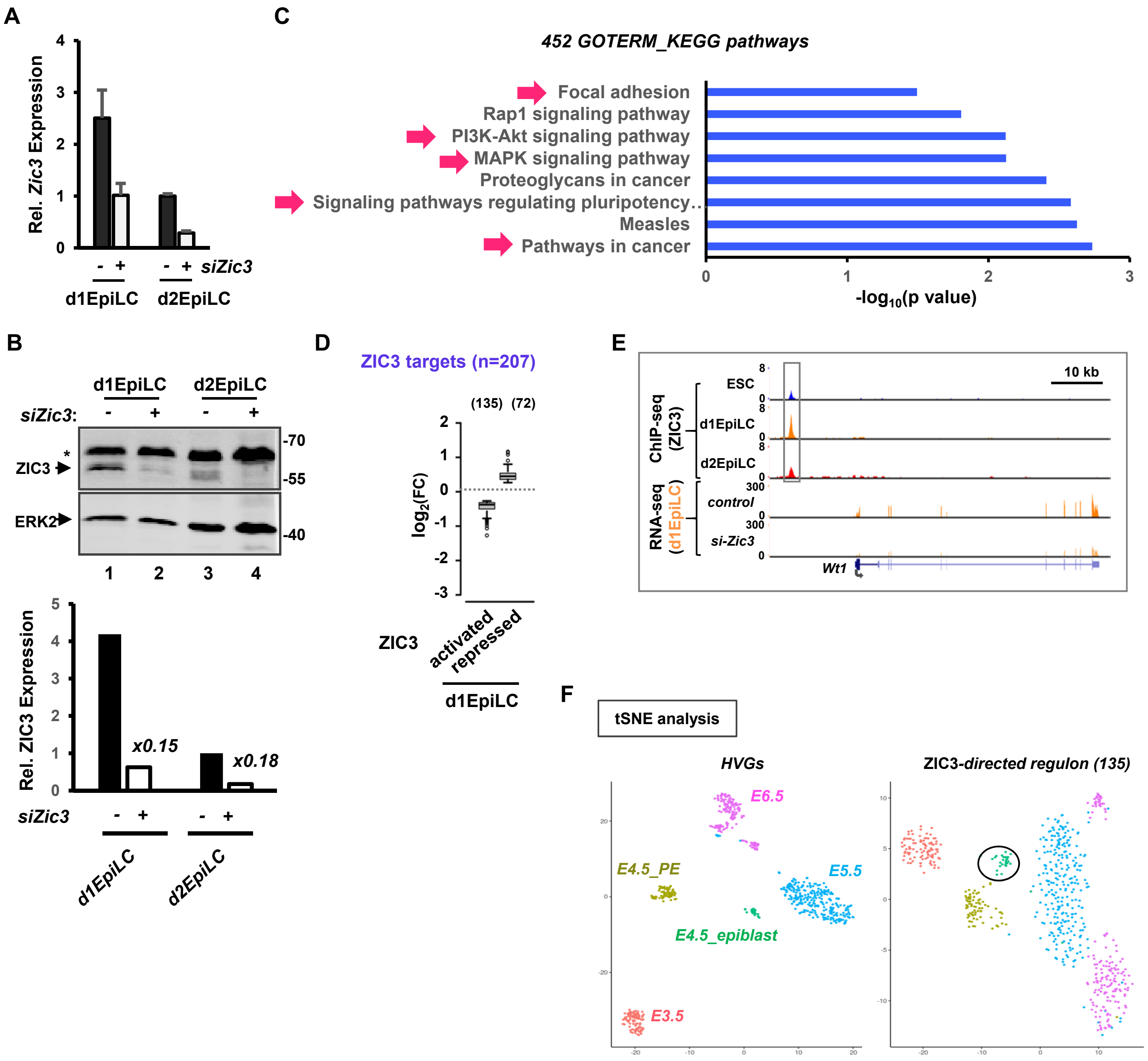

Figure S8. Properties of ZIC3-regulated genes. Related to Figures 4 and 5. (A and B) siRNA-mediated depletion of ZIC3. Levels of *Zic3* mRNA in d1EPiLCs and d2EpiLCs were measured by RT-qPCR (n=3) (A) and ZIC3 protein by western blot analysis (B). RT-qPCR data are shown relative to *Zic3* levels in d2EpiLCs treated with non-targeting control (-). ERK2 protein levels are shown as a loading control. The asterisk represents a cross reacting non-specific band. The protein expression data are quantified in the graph below and the fold change in expression caused by *Zic3* depletion is indicated. (C) Gene ontology analysis of ZIC3-regulated genes for the KEGG pathways. (D) Boxplot showing the overall changes in expression of genes directly bound by ZIC3 following *Zic3* depletion with siRNA in d1EpiLCs. The total number of genes going down (ie activated by ZIC3) and going up (ie repressed by ZIC3) is indicated above the graph. (E) UCSC genome browser views of the ZIC3 ChIP-seq (top) and RNA-seq (bottom) profiles around the *Wt1* locus. The major ZIC3 binding peak is boxed. (F) t-SNE analysis of scRNA-seq data from mouse embryos (Mohammed et al., 2017) using the HVGs from all of the scRNAseq data (left) or the 135 directly activated ZIC3 target genes (right). The colour coding from the HVG-derived t-SNE analysis and the cluster comprised of E4.5 cells is circled.

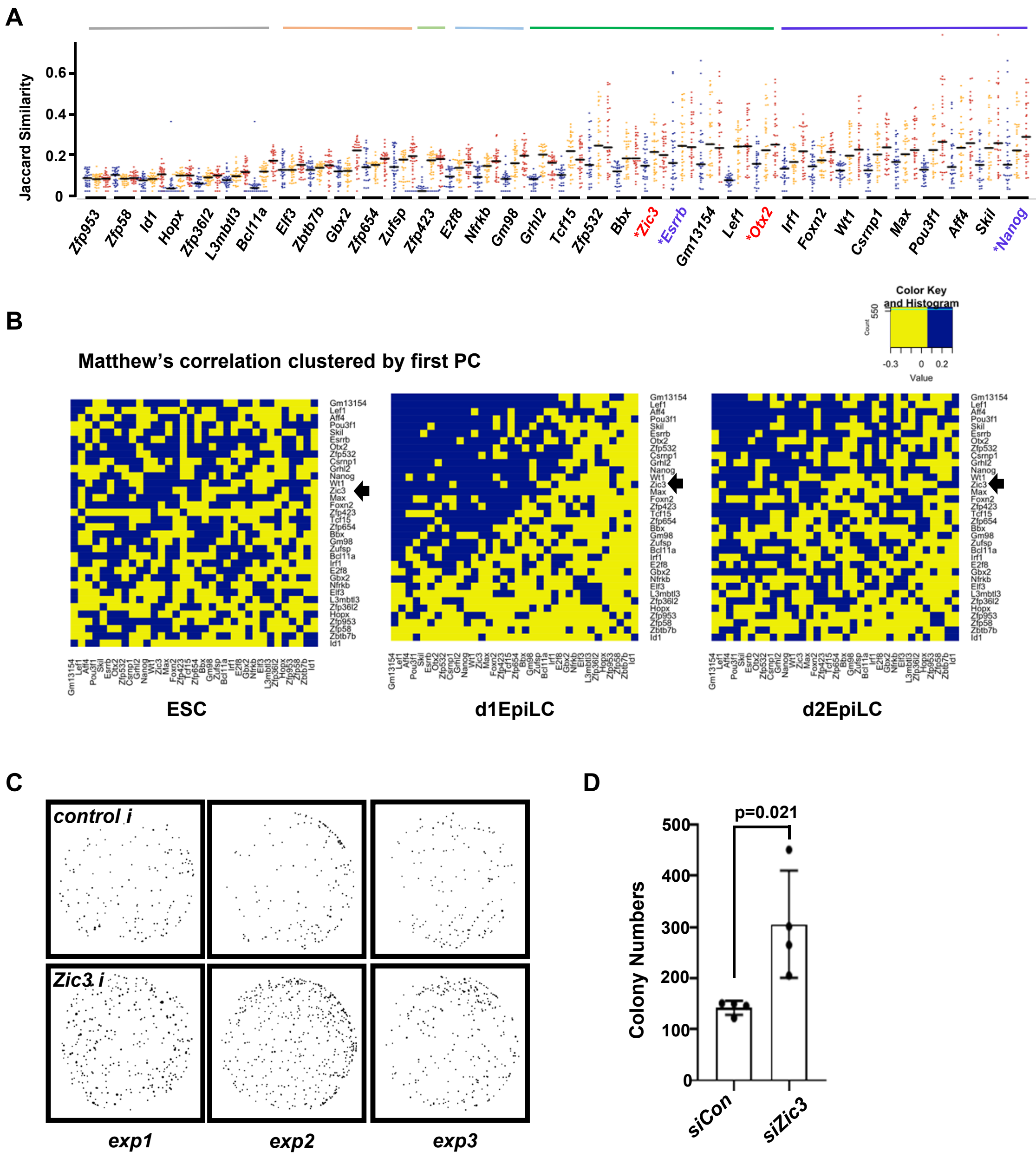

Figure S9. Co-expression analysis of ZIC3 activated transcription factors and phenotypic outcome of *Zic3* depletion. Related to Figure 6. (A) Jaccard's similarity score for each of the indicated ZIC3 activated target genes (in black font) to each other in ESCs (blue), d1EpiLCs (orange) and d2EpiLCs (Red). Black lines represent the median similarity scores. Only 31 target genes that encode transcription factors were included in this cross-comparison. The majority of genes exhibit higher similarity scores in d1EpiLCs which is often maintained in d2EpiLCs. (B) Matthew's correlation plots of the co-expression of the indicated transcription factor-encoding ZIC3 activated genes in ESCs (left), d1EpiLCs (middle) and d2EpilCs (right). The location of *Zic3* is highlighted with an arrow. (C and D) ESC clonogenicity assay. (C) Alkaline phosphatase-positive colonies were detected in ESCs grown in EpiLC differentiation media for 15 hours and returned to 2i/LIF medium. Cells were treated with either control siRNAs (top) or siRNAs targeting *Zic3* (bottom). (D) Quantification of the experiments showing individual data points from four independent experiments.
